# Attention Deficit Hyperactivity Disorder following Hypothalamic Hamartoma Surgery : An unusual manifestation

**DOI:** 10.1192/j.eurpsy.2022.1115

**Published:** 2022-09-01

**Authors:** A. Khivsara, D. Goya, N. Nebhinani

**Affiliations:** 1 All India Institute of Medical Sciences, New Delhi, Psychiatry, New Delhi, India; 2 Jeevan Dhara Multispeciality hospital, Psychiatry, Sumerpur(Pali), India; 3 All India Institute of Medical Sciences, Jodhpur, Psychiatry, Jodhpur, India

**Keywords:** Hypothalamic hamartoma, risperidone, epilepsy, adhd

## Abstract

**Introduction:**

Psychiatric symptoms are a common comorbid feature of hypothalamic hamartoma(HH) with epilepsy. They are a significant challenge for patient and their families. Most common psychiatric symptoms are externalizing behaviors such as aggression and defiance.

**Objectives:**

To outline an atypical presentation of HH in form of development of ADHD post-surgery.

**Methods:**

A 6-year old child born out of non-consanguinous marriage, with history of hyperemesis gravidarum and depression in mother in ante-natal period, delivered by NVD at term(did not cry at birth and was hospitalized for 3 days) with birth weight of 2.25 kg, currently presented to Neurology with global developemental delay and history of gelastic seizures since 3 years of age. Patient was diagnosed with pituitary hamartoma(through MRI) and precocious puberty that time and was operated for it after which he started having behavioural issues like irritability, aggression, hyperactivity and lack of appropriate social behaviour with peers along with defiance towards parents. Child was then refered to Psychiatry. On MSE patient did not interact with interviewer and was noticed to shout loudly when confronted for using mobile phone. MRI brain(2 months back) showed post-op changes with cystic lesion in suprasellar region. IQ assessment showed borderline intelligence.

**Results:**

Patient was started on Risperidone(upto 1.5 mg) which lead to some improvement. However antiepileptics are being rationalized to prevent behavioural issues secondary to epilepsy

**Conclusions:**

Patients of HH with epilepsy, present with varied psychiatric symptoms which usually improve after surgery. However we came across a child with worsening of psychiatric symptoms after he was operated for above lesion.

**Disclosure:**

No significant relationships.

